# Physiological Effects of Double Ovariotomy

**Published:** 1873

**Authors:** 


Physiological Results of Double Ovariotomy.—Kœberle, of Strasbourg, writes to M. Puech (*Montpellier Medical*) that his patients on whom he has performed the operation of double ovariotomy are not less loving to their friends, parents or husbands. The genital organs remain excitable. Their character has become sweeter and less irascible. The breasts have not diminished. There is no tendency to obesity where it has not already existed; no alteration in the increase of hair, nor modification of the voice. Perfect health has been the rule in spite of cessation of the menses. The patients do not lose their distinctive characters.

